# Cost Savings of Expedited Care with Upfront Next-Generation Sequencing Testing versus Single-Gene Testing among Patients with Metastatic Non-Small Cell Lung Cancer Based on Current Canadian Practices

**DOI:** 10.3390/curroncol30020180

**Published:** 2023-02-15

**Authors:** Brandon S. Sheffield, Kiefer Eaton, Bruno Emond, Marie-Hélène Lafeuille, Annalise Hilts, Patrick Lefebvre, Laura Morrison, Andrea L. Stevens, Emmanuel M. Ewara, Parneet Cheema

**Affiliations:** 1 William Osler Health System, Brampton, ON L6R 3J7, Canada; 2 Janssen, Inc., Toronto, ON M3C 1L9, Canada; 3 Analysis Group, Inc., Montréal, QC H3B 0G7, Canada; 4 Janssen Global Services, Inc., Raritan, NJ 08869, USA

**Keywords:** non-small cell lung cancer, economic model, next-generation sequencing, testing costs, medical costs

## Abstract

This study assessed the total costs of testing, including the estimated costs of delaying care, associated with next-generation sequencing (NGS) versus single-gene testing strategies among patients with newly diagnosed metastatic non-small cell lung cancer (mNSCLC) from a Canadian public payer perspective. A decision tree model considered testing for genomic alterations using tissue biopsy NGS or single-gene strategies following Canadian guideline recommendations. Inputs included prevalence of mNSCLC, the proportion that tested positive for each genomic alteration, rebiopsy rates, time to test results, testing/medical costs, and costs of delaying care based on literature, public data, and expert opinion. Among 1,000,000 hypothetical publicly insured adult Canadians (382 with mNSCLC), the proportion of patients that tested positive for a genomic alteration with an approved targeted therapy was 38.0% for NGS and 26.1% for single-gene strategies. The estimated mean time to appropriate targeted therapy initiation was 5.1 weeks for NGS and 9.2 weeks for single-gene strategies. Based on literature, each week of delayed care cost CAD 406, translating to total mean per-patient costs of CAD 3480 for NGS and CAD 5632 for single-gene strategies. NGS testing with mNSCLC in current Canadian practice resulted in more patients with an identified mutation, shorter time to appropriate targeted therapy initiation, and lower total testing costs compared to single-gene strategies.

## 1. Introduction

Lung cancer (LC) is the most commonly diagnosed cancer in Canada and remains the leading cause of cancer-related deaths [1]. Non-small cell LC (NSCLC) accounts for nearly 90% of all histologically confirmed cases of LC in Canada [2]. Patients with advanced or metastatic NSCLC (mNSCLC) are suitable candidates for targeted therapies depending on the presence of actionable genomic alterations in driver oncogenes, such as *EGFR, ALK, ROS1,* and *BRAF* [3]. Evidence suggests that personalized treatment of mNSCLC guided by routine molecular profiling results in significant improvements in clinical outcomes [4]. As such, biomarker testing has become an integral component of the standard of care for Canadian patients with mNSCLC [5].

Testing strategies currently available in Canada for the identification of driver mutations in mNSCLC comprise single-gene testing, such as sequential or exclusionary testing, and multi-gene testing, such as next-generation sequencing (NGS). Relative to single-gene testing, NGS has been shown to lead to the correct identification of more actional genomic alterations, which conferred a greater benefit from targeted therapies and improved the treatment response [6,7,8]. Use of a single NGS test also reduces delays in the time taken to test results and the need for retesting and repeat biopsies [9,10]. Additionally, as NGS enables upfront detection of non-standard mutations in NSCLC, this testing strategy can also inform clinical trial eligibility for patients with alterations without currently approved targeted therapies [7,8,11].

A recent health and budget impact model among Canadian patients with NSCLC (with Ontario as a sample province) showed that relative to a scenario considering 100% of testing with single-gene testing strategies, a 50% uptake of NGS was associated with an increase in the proportion of patients initiating appropriate targeted therapies, leading to a projected 3-year gain of 680.9 life years [6]. The estimated budget impact of CAN 37.1 million translated to CAN 0.87 per Ontario resident per year [6], suggesting NGS can optimize targeted treatment selection without imposing a substantial economic burden on the Canadian healthcare system.

Previous studies among patients with mNSCLC in the United States (US) have found that per-patient testing costs are lower for those undergoing testing with NGS relative to single-gene testing strategies [12,13,14]; however, there is a paucity of research evaluating the total cost of testing in Canada that incorporates the medical costs associated with testing, such as oncologist visits and costs of rebiopsies including related complications. In addition, given that NGS has been found to be associated with a shorter time to initiation of appropriate targeted therapy [14], an evaluation of the estimated costs associated with delaying care is warranted. To this end, the current study developed an economic model to evaluate the total costs of testing, including estimated costs of delaying systemic therapy, associated with NGS versus single-gene testing strategies among patients with mNSCLC from a Canadian public payer perspective.

## 2. Materials and Methods

### 2.1. Model Framework

In order to evaluate the total cost of testing, including the estimated costs of delaying care, associated with NGS versus single-gene testing strategies, a decision tree model was developed. The model included the time from the first test after a diagnosis of mNSCLC until biomarker test results were achieved and appropriate targeted therapy was subsequently initiated. A Canadian population of publicly insured adult mNSCLC patients whose genomic status was unknown and who were treatment-naïve were included in the model. The study used Excel 2016 software (Microsoft Corporation, Redmond, WA, USA).

### 2.2. Model Structure

Patients with a diagnosis of mNSCLC received an initial biopsy upon model entry, similar to previous decision analytic models evaluating testing costs of NGS in this population [12,14]. Subsequently, patients were tested using NGS or an alternative single-gene testing strategy (i.e., exclusionary, sequential, non-comprehensive sequential, or rapid panel testing), defined using the following sequences:

#### 2.2.1. NGS

Tissue biopsy was used to perform a broad spectrum biomarker panel that followed clinical guidelines to simultaneously test for recommended alterations (*EGFR, ALK, ROS1, BRAF, KRAS, MET, HER2, RET*, *NRG1,* and *NTRK1/2/3*) [5]

#### 2.2.2. Exclusionary

A test for KRAS (the most frequently observed mutation in non-squamous NSCLC in Canada) [5] was considered first, since genetic alterations are assumed to be mutually exclusive. A positive test result ended the testing sequence, while sequential testing (as included below) was conducted following a negative test result.

#### 2.2.3. Sequential

A sequence of single-gene tests for alterations in the order of *EGFR, ALK*, then *ROS1* based on recommendations in clinical guidelines [5] whereby any positive result ended the testing sequence, and negative tests for all three alterations resulted in a sequence of single-gene tests in the following order: *BRAF, KRAS, MET, HER2, RET, NRG1,* and *NTRK1/2/3* (assuming the prior alteration received a negative result).

#### 2.2.4. Non-Comprehensive Sequential

A sequence of single-gene tests for alterations in the order of *EGFR, ALK,* then *ROS1* based on clinical guideline recommendations [5] whereby the sequence ended after testing for the three alterations, irrespective of the test results.

#### 2.2.5. Rapid Panel

Simultaneous single-gene tests for alterations *EGFR, ALK,* and *ROS1* based on recommendations in clinical guidelines [5] whereby a positive result ended the testing sequence, and negative tests for all three alterations resulted in simultaneous single-gene tests for the other alterations (i.e., *BRAF, KRAS, MET, HER2, RET, NRG1,* and *NTRK1/2/3*).

Further testing may have been performed if a patient’s genomic alteration was not initially identified, which may have necessitated a rebiopsy if there was insufficient tumor tissue or DNA remaining.

The decision tree model ended with the initiation of appropriate targeted therapy following a correctly identified genomic alteration. For the duration of the model, the total cost of testing, which included testing costs, costs of rebiopsies and associated complications, interventional radiology visits, and oncologist visits, was evaluated for each testing strategy. In addition, the total cost of testing including estimated costs associated with delaying care, based on a Canadian-specific literature-based estimate of costs in the pre-diagnosis phase [15] and the time to initiation of appropriate targeted therapy, was evaluated for each testing strategy.

### 2.3. Model Assumptions

The following assumptions (based on literature, public data, and expert opinion) were incorporated:

1. Irrespective of the testing strategy, all patients underwent PD-L1 immunohistochemistry testing.

2. The model assumed each patient followed only one testing strategy, and all eligible patients were tested.

3. The proportion of patients tested with each strategy for the base case was 50% for NGS based on the available literature [6,16,17], and 5% for sequential, 10% for exclusionary, 25% for non-comprehensive sequential, and 10% for a rapid panel based on expert opinion.

4. DNA+/−RNA-based NGS was considered; combined DNA- and RNA-based testing strategies which may be used to identify gene fusions were not considered.

5. The mutation-specific rate of positive detection was consistent for all testing strategies and was based on the available literature [6,18,19,20,21].

6. At the beginning and end of the testing sequence, patients received one oncologist visit to discuss biopsy test results and another oncologist visit to discuss steps for appropriate targeted therapy initiation.

7. Patients using single-gene tests who tested negative for *EGFR* and *ALK* mutations received an oncologist visit before continuing the testing sequence.

8. Across all testing strategies, among patients requiring a rebiopsy, 30% were assumed to receive one [22] and had an interventional radiology visit for the procedure.

### 2.4. Model Inputs

#### 2.4.1. Population Inputs

The population included a hypothetical cohort of 1,000,000 publicly insured Canadian adult patients to ascertain the budget impact on the Canadian healthcare system, consistent with existing budget impact analyses in advanced NSCLC [23,24,25]. The Canadian Cancer Statistics [2] were used to estimate the proportion of adults with LC, and among them, the proportions with NSCLC and mNSCLC (Table 1).

#### 2.4.2. Clinical Inputs

Expert opinion was used to determine the proportion of patients receiving testing with each strategy. The rate of positive genomic identification, the time to test results, the time to oncologist visit, and the time to rebiopsy were literature based. Additionally, literature-based estimates were used for the proportion of patients who required and received rebiopsy, the proportion who failed rebiopsy, and the proportion with rebiopsy complications (Table 1).

#### 2.4.3. Cost Inputs

Testing costs for each strategy were based on the Ontario’s University Health Network (UHN) Laboratory Medicine Program costs, which included sample processing and handling costs [29]. Medical costs associated with testing, including interventional radiology and oncologist visit costs, rebiopsy and related complication costs, PD-L1 testing costs, and estimated costs associated with delaying care were based on the Ontario Schedule of Benefits [31], the Canadian Institute for Health Information [32], or a targeted literature review (Table 1).

### 2.5. Model Outputs

Model outputs for each testing strategy included the proportion of patients who tested positive for a genomic alteration with an approved targeted therapy, the time to initiation of appropriate targeted therapy (i.e., time to test results [mutation specific] + clinical turnaround time to see an oncologist (2 weeks)), and the total costs per patient tested from the first test following a diagnosis of mNSCLC until appropriate targeted therapy initiation (Table 2). All costs were estimated from a Canadian public payer perspective, with total costs per patient reported for all single-gene testing strategies combined, as well as for each specific testing strategy (i.e., sequential, exclusionary, non-comprehensive sequential, and rapid panel). Per-patient total costs included total testing costs (i.e., testing costs and testing-related medical costs) and total costs of care, which also included estimated costs associated with delaying care. Costs associated with delayed care were based on an analysis of patients with LC that reported mean per-patient costs during the 3-month pre-diagnosis phase using Ontario provincial registry and administrative data [15]. Weekly costs were estimated by dividing the 3-month estimate by 12; estimated costs associated with delayed care were evaluated by multiplying the weekly cost by the time to initiation of appropriate targeted therapy that was evaluated for each testing strategy. Cost values were reported in 2021 CAD, inflated based on the health care services component of the Statistics Canada Consumer Price Index [33].

### 2.6. Impact of Increasing the Proportion of Patients Tested with NGS

To evaluate the budget impact of increasing the use of NGS testing, the base case distribution of NGS and single-gene testing utilization (i.e., model assumption #3) was compared to a scenario where the proportion of patients receiving NGS testing was increased from 50% to 70%, with reduced proportions receiving testing with single-gene strategies (i.e., 5% sequential, 5% exclusionary, 15% non-comprehensive sequential, and 5% rapid panel). The estimated budget impact was evaluated using the updated distribution of testing strategy utilization and the cost per patient associated with each testing strategy. In addition, the clinical impact was evaluated, comparing the proportion of patients testing positive for an actionable mutation with an approved targeted therapy and the time to initiation of appropriate targeted therapy based on the initial and modified distribution of testing strategy utilization.

### 2.7. Sensitivity Analysis

Using a one-way sensitivity analysis (OSA), the robustness of results was assessed by individually varying selected model inputs (e.g., rates of positive identification for EGFR and KRAS mutations, rebiopsy rates, testing costs, oncologist visit costs, interventional radiologist visit costs, and estimated costs associated with delaying treatment) to determine the impact of each parameter on the estimated costs for NGS and the combination of all four single-gene testing strategies (Table 3). Literature published in the past 5 years, or a pre-specified threshold (e.g., 20% above or below the base case) was used to assign ‘high’ and ‘low’ values to model inputs, which corresponded to the identified upper and lower bounds.

## 3. Results

Among 1,000,000 hypothetical publicly insured adult Canadians, a total of 382 patients were estimated to be eligible for genetic testing based on the presence of a diagnosis for mNSCLC. A higher proportion of patients tested positive for a genomic alteration with an approved targeted therapy using NGS (38.0%) than single-gene testing strategies (total 26.1%: 31.7% sequential, 29.3% exclusionary, 20.3% non-comprehensive sequential, and 34.4% rapid panel). Patients tested with NGS had the shortest estimated mean time to initiation of appropriate targeted therapy (5.1 weeks) compared to all single-gene testing strategies (total 9.2 weeks: 16.1 sequential, 15.3 exclusionary, 6.4 non-comprehensive sequential, and 7.0 rapid panel (Table 2)).

### 3.1. Total Cost for Patients Undergoing Testing with NGS versus Single-Gene Testing Strategies

Patients receiving NGS testing had the lowest total cost of testing at CAD 3480 per patient versus CAD 5632 for all single-gene testing strategies combined, with CAD 9044 for sequential, CAD 8533 for exclusionary, CAD 3745 for non-comprehensive sequential, and CAD 5740 for rapid panel, which included the estimated costs associated with delaying care (Table 2). The cost of testing at the population level totaled CAD 663,998 for NGS relative to CAD 1,075,222 for all single-gene testing strategies combined.

**Figure 1:**
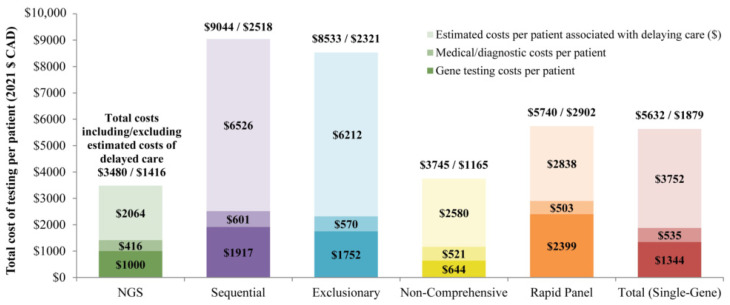
Total cost of testing per patient by strategy, including/excluding estimated costs of delayed care ^a,b^. **Abbreviations:** NGS = next-generation sequencing. **Notes**: a. Total costs include gene testing costs, testing-related medical costs, and estimated costs of delayed care. The estimated costs associated with delaying treatment were calculated as the time to initiation of appropriate targeted therapy times estimated weekly cost during the pre-diagnosis phase. b. Costs include gene testing costs, rebiopsy costs, costs for interventional radiologist and oncologist visits, and PD-L1 testing costs.

Excluding the estimated costs associated with delaying care, the total per patient testing and testing-related medical costs were CAD 1416 for NGS versus CAD 1879 for all single-gene testing strategies combined, with CAD 2518 for sequential, CAD 2321 for exclusionary, CAD 1165 for non-comprehensive sequential, and CAD 2902 for rapid panel (Table 2). The cost of testing at the population level totaled CAD 270,117 for NGS relative to CAD 358,773 for all single-gene testing strategies combined. Medical costs associated with testing and PD-L1 immunohistochemistry diagnostic services accounted for 29% of the total costs of NGS, 23% of sequential, 24% of exclusionary, 45% of non-comprehensive sequential, and 28% of rapid panel; the remaining proportion was attributable to genetic testing costs (Figure 1).

### 3.2. Impact of Increasing the Proportion of Patients Tested with NGS

**Figure 2:**
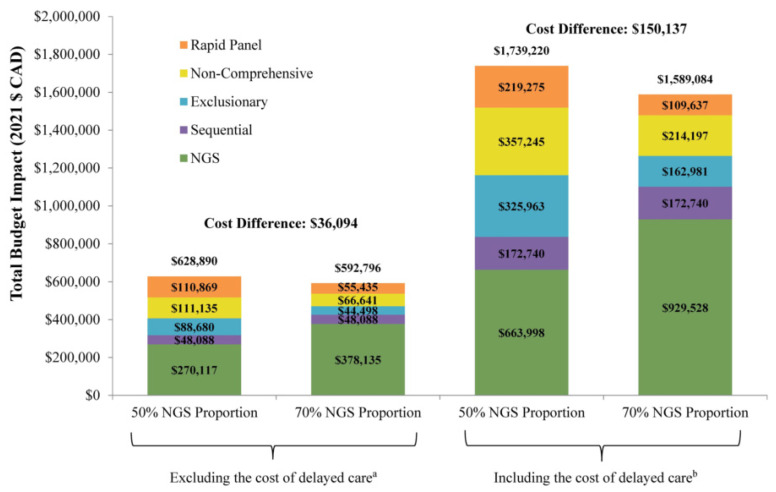
Total budget impact of increasing the proportion of patients tested with NGS from 50% to 70%. **Abbreviations:** NGS = next-generation sequencing. **Notes**: a. Costs include gene testing costs, rebiopsy costs, costs for interventional radiologist and oncologist visits, and costs of PD-L1 testing. b. Total costs include genetic testing costs, medical costs associated with testing, and estimated costs of delayed care. The estimated costs associated with delaying treatment were calculated as the time to initiation of appropriate targeted therapy times estimated weekly cost during the pre-diagnosis phase.

Upon revising the distribution of testing strategy utilization to increase the proportion of patients tested with NGS to 70% with 5% sequential, 5% exclusionary, 15% non-comprehensive sequential, and 5% rapid panel, the incremental budget impact resulted in savings of CAD 0.013 per-member-per-month (PMPM; Table 4), translating to cost savings of CAD 150,137 per year to the Canadian public healthcare system (Figure 2). Excluding estimated costs associated with delaying care, cost savings were CAD 0.003 PMPM and CAD 36,094 at the plan level. Extrapolating results to the entire Canadian adult population of 30.2 million residents [37], potential savings could reach CAD 4,534,137, assuming the same initial and modified distribution of testing strategy utilization among the projected number of Canadian adults with mNSCLC. In addition to the cost savings, increasing the proportion of patients tested with NGS resulted in 34.4% of patients testing positive for an alteration with an approved targeted therapy, an increase from 32.0% in the base case single-gene testing scenario. Furthermore, the overall mean time to initiation of appropriate targeted therapy decreased from 7.2 to 6.5 weeks per patient.

### 3.3. Sensitivity Analysis

**Figure 3:**
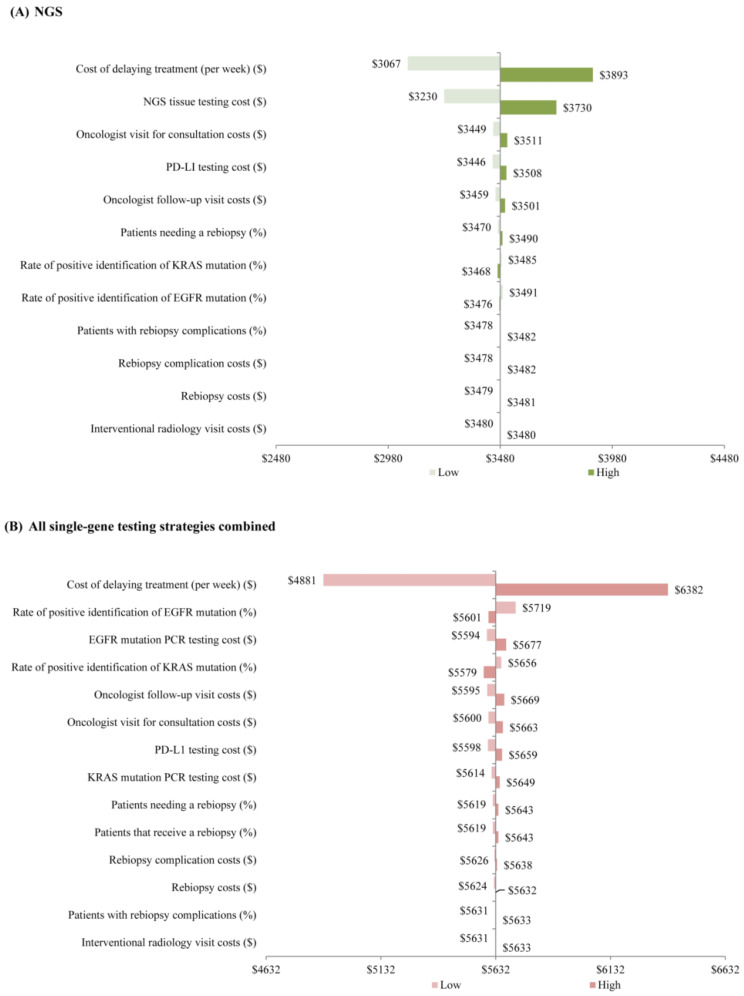
Sensitivity analysis (**A**) among patients undergoing NGS testing and (**B**) for all single-gene testing strategies combined ^a^. **Abbreviations:** NGS = next-generation sequencing. **Note**: a. Published literature or a specified threshold of ±20% was used to allocate low/high values used for the inputs in the sensitivity analysis (inputs defined in Table 3).

Including the estimated costs associated with delaying care, the total cost of testing for those undergoing NGS testing ranged between CAD 3067 and CAD 3893 per patient, which was consistently lower than all single-gene testing strategies combined (CAD 4881 to CAD 6382 per patient). The estimated weekly cost associated with delaying care, NGS tissue testing costs, and oncologist visit costs were the model inputs with the largest impact on the total cost of NGS (Figure 3A). Model inputs with the largest influence on the total cost of testing for the combined single-gene testing strategies were the estimated weekly cost associated with delaying care, the rate of positive identification of an EGFR mutation, and EGFR mutation testing costs (Figure 3B).

## 4. Discussion

This study used a decision tree model to evaluate the total costs of testing among newly diagnosed Canadian adult patients with mNSCLC undergoing testing with NGS versus single-gene testing strategies, including testing costs, medical costs associated with testing, and estimated costs of delaying care. Relative to alternative single-gene testing strategies, NGS was associated with the lowest total cost of testing per patient, including costs related to delayed care, and resulted in substantial cost savings from the Canadian public payer’s perspective. Additionally, broad molecular profiling with NGS led to the identification of the largest proportion of patients testing positive for an actionable mutation with an approved targeted therapy and was associated with the shortest time to initiation of appropriate targeted therapy.

Several studies have reported on the utility, cost-effectiveness, and budget impact of multi-gene testing as an alternative to single-gene testing in patients with mNSCLC worldwide [38], including Canada [6,30,39], the US [12,13,14], Europe [4], and East Asia [40]. In Canada, a recent budget impact model among patients with mNSCLC showed that introducing a 50% uptake of NGS, relative to 100% of testing with single-gene testing strategies, was associated with 680.9 incremental life years at an estimated budget impact of CAD 37.1 million over a 3-year period [6]. The current model adds value by providing a more comprehensive estimate of the total cost of testing for patients undergoing testing with NGS and single-gene testing strategies, given the incorporation of medical costs, including rebiopsy and associated complications, as well as the costs of visiting oncologists and interventional radiologists. Assuming patients with mNSCLC undergo molecular profiling using NGS, the per-patient total testing costs, excluding the estimated costs of delaying care, were CAD 1416 relative to CAD 1879 for single-gene strategies. These cost saving trends are consistent with those observed in payer systems in the US, including Medicare and commercial perspectives [14]. However, in countries in which the prevalence of genomic alterations and current testing and reimbursement strategies differ, such as in Hong Kong, other testing strategies may be more cost-saving [41].

The current analysis also provides an estimate of the costs associated with delayed care. Prior work using Ontario provincial registry and administrative data found mean per-patient costs of CAD 4870 (2021 CAD) during the 3-month pre-diagnosis phase, which translated to an estimated cost of CAD 406 for each week of delayed care, including costs of inpatient hospitalization and surgery, physician services, diagnostic tests, prescription drugs, and home and community care [15]. The estimated weekly cost is consistent yet lower than prior work evaluating physician and hospital costs associated with delayed wait times among patients diagnosed with NSCLC between 1996 and 2000 in Manitoba, which found per-patient costs of CAD 13,565 (2021 CAD) during the 6-month pre-diagnosis phase, equating to an estimated weekly cost of CAD 522 [42]. For patients with mNSCLC tested with NGS, the mean time to initiation of appropriate targeted therapy was 5.1 weeks translating to estimated costs of delaying care of CAD 2064 per patient, relative to 9.2 weeks and estimated costs amounting to CAD 3752 for patients tested with single-gene testing strategies. Including testing and testing-related medical costs, increasing the proportion of patients using NGS from 50% to 70% resulted in potential savings of CAD 4,534,137 in a pan-Canadian context, a 2.4% increment of patients testing positive for an alteration with an approved targeted therapy, and decreased the mean time to initiation of appropriate targeted therapy by nearly 1 week (7.2 weeks versus 6.5 weeks). These results may improve overall survival (OS), given that therapy initiation, including chemotherapy and specifically targeted therapy, has been found to be associated with improved OS [43,44,45,46]. Specifically, prior research has found that delaying NSCLC treatment by as little as one week can result in a 4% increase in mortality [47]. Prior research using data from the National Cancer Data Base in the US found that among patients with advanced mNSCLC, forgoing treatment was associated with significantly lower median OS of 2.0 months relative to 9.3 months among patients who received chemotherapy [43]. Furthermore, among patients with *EGFR*-mutated mNSCLC, the receipt of targeted therapies relative to chemotherapy has been associated with numerically higher median OS (22.9 months versus 19.5 months) [44], and patients receiving next-generation relative to first-generation therapies have demonstrated significantly higher median OS (38.6 months versus 31.8 months) [45]. Consistent with prior findings [14], the current model shows that patients tested using NGS receive appropriate therapy an average of 4 weeks faster than those using other single-gene testing strategies (5.1 weeks versus 9.2 weeks); the time to appropriate targeted therapy initiation is estimated to be as long as 15–16 weeks for patients undergoing exclusionary and sequential testing. By allowing for the simultaneous screening of multiple mutations, NGS has the potential to meaningfully reduce treatment delays, which could lead to cost savings and improved survival outcomes.

Evidence supports the use of multi-gene testing as a reliable sequencing tool for the detection of mutations, including in advanced cancer [7,48], with prior research reporting that among patients with NSCLC who had previously been tested and deemed negative for *EGFR* and *ALK* mutations using single-gene strategies, 17% and 35% were identified as positive using NGS testing, respectively [49,50]. Furthermore, a real-world sample of 2316 patients reported that single-gene polymerase chain reaction testing was projected to miss up to 50% of patients who tested positive for actionable *EGFR* Exon20 insertion mutations using NGS [51]. In addition to *EGFR* and *ALK* mutations, existing evidence has demonstrated improved detection rates for other commonly tested genomic alterations [9]. In a study of patients with LC who had previously tested negative using single-gene testing strategies for alterations in 11 commonly tested genes, NGS was able to identify genomic alterations with an approved targeted therapy among 26% of patients, and an additional 39% of patients who had a genomic alteration with a targeted agent available in a clinical trial [9]. In the current study, 38% of patients with mNSCLC using NGS tested positive for an actionable genomic alteration with an approved targeted therapy relative to only 26% with alternative testing strategies. Previous studies have reported the advantages of targeted therapies relative to chemotherapy for vulnerable patient populations with advanced cancers, including more favorable clinical outcomes and safety profiles [7,52,53]. Given the improvements in survival associated with genotype-driven targeted therapies for patients with mNSCLC, they continue to be a key element of disease management for this population, guided by broad molecular profiling. Together, these findings underscore the value of NGS as a diagnostic strategy to identify patients with actionable mutations who can benefit from approved targeted therapies, or as a means of facilitating enrollment in clinical trials for treatments under development.

In Canada, there is currently no national oversight for genomic profiling, and reimbursement strategies vary across provinces [5,54]. The incongruity in funding and clinician awareness on broad genomic profiling has contributed to country-wide variation in the proportion of patients with mNSCLC tested for genomic alterations, with rates as low as 10% in some areas [55]. In addition, opportunities for other testing strategies, such as reflex testing whereby pathologists may continue testing subsequent to an inconclusive primary test result, may lead to more efficient testing and reduce the time to initiation of appropriate targeted therapy. However, barriers to implementing reflex testing in real-world practice in Canada include limited awareness, the need for funding in Canadian public healthcare systems, and the necessary coordination between the oncologists, pathologists, and specialists acquiring the tissue sample [28]. With the continuous discovery of new driver mutations and the development of novel targeted therapies, broad molecular testing continues to be recommended for patients with mNSCLC to guide disease management, yet the lack of funding presents a barrier to wider use of NGS in clinical practice, such that some clinics may continue using cheaper alternatives. For example, while the estimated per-patient testing and testing-related medical costs were CAD 1165 for non-comprehensive sequential testing versus CAD 1416 for NGS, only 20% of patients tested positive for an actionable mutation relative to 38% of patients tested with NGS. The need for repeated sample collection to test relevant biomarkers using single-gene testing strategies may additionally impose a burden on smaller testing centers that lack in-house laboratories. Conversely, comprehensive profiling precludes the need for additional testing in the clinical setting [5,11,39,40,56]. While the current study did not evaluate societal costs (e.g., complex infrastructure, equipment, and labor), these costs merit consideration in future analyses as they may pose a barrier to clinical adoption in regional settings [30]. Given that broad genomic profiling with NGS has the potential to reduce treatment delays, policies aimed at allocating and standardizing funding across Canada warrant consideration.

### Limitations

These findings should be interpreted in light of certain limitations. First, the results reported in this study are based on an economic model that applied a decision tree and budget impact structure. As such, some of the assumptions or inputs incorporated into the model were based on expert opinion or restricted to published literature data availability and may contain uncertainty. For example, the time-to-test results may vary by testing center. While requisite analysis of real-world data, including an evaluation of alternative strategies for genetic testing employed in Canadian clinical practice, should precede definitive conclusions, results of the current model remained robust in the sensitivity analyses. Second, the amount reimbursed for testing was estimated based on Ontario’s UHN programming costs, and NGS testing costs did not consider the cost of marketed commercial assays; therefore, the results may not be generalizable to other provinces or smaller regional testing centers. Furthermore, generalizability to countries other than Canada may be limited by differences in disease estimates, payer structures, reimbursement policies, as well as testing and medical costs. Third, though the work of De Oliveira et al. does not directly provide the cost of delaying treatment, an estimate of CAD 406 was used as a proxy for the cost of each week of delayed care. This estimate was based on a population of patients in Ontario, and similar prior evidence on the expenses incurred due to delayed wait times prior to receipt of a confirmatory diagnosis was found among patients in Manitoba [42]. Fourth, the source of the specimen for testing was not considered in the model, and rebiopsy costs were based on core needle biopsies. Additional research is needed to determine the impact of other sources of specimens (e.g., through endobronchial ultrasound) on testing costs. Therapy costs were also not included in the model, which would allow for an estimate of a broader range of costs and warrants future research. Finally, clinical and pathological data related to the histological subtype of carcinoma or smoking history were not considered for the hypothetical population of patients with mNSCLC included in the model which would potentially influence the proportion of patients testing positive for a genomic alteration with an approved targeted therapy; further work is required to assess the total cost of testing among patients with specific tumor subtypes and clinical profiles.

## 5. Conclusions

Relative to single-gene testing strategies, this decision tree model found that NGS testing among Canadian adults newly diagnosed with mNSCLC resulted in a higher proportion of patients testing positive for an alteration with an approved targeted therapy, the fastest time to initiation of appropriate targeted therapy, and the lowest total testing cost per patient. Cost savings were consistently observed when model inputs were varied in sensitivity analyses. Policies that aim to allocate and standardize funding for NGS across Canada warrant consideration.

## Figures and Tables

**Table 1:** Summary of model inputs.

Population and Clinical Inputs		Value	Source
Population	Number of covered lives	1,000,000	Assumption
	% of adults with LC	0.066%	Canadian Cancer Statistics, 2020 [2]
	% with NSCLC among LC population	88.0%	Canadian Cancer Statistics, 2020 [2]
	% diagnosed metastatic, Stage III and Stage IV, among NSCLC population	65.7%	Canadian Cancer Statistics, 2020 [2]
Testing strategy distribution	% undergoing testing with tissue NGS	50.0%	[6,16,17]
	% undergoing sequential testing	5.0%	Expert Opinion
	% undergoing exclusionary testing	10.0%	Expert Opinion
	% undergoing non-comprehensive testing	25.0%	Expert Opinion
	% undergoing rapid panel testing	10.0%	Expert Opinion
Frequency of genomic alterations	% testing positive for EGFR mutation ^a^	17.0%	Johnston et al., 2020 [6]
	% testing positive for ALK mutation ^a^	3.0%	Johnston et al., 2020 [6]
	% testing positive for ROS1 mutation ^a^	1.0%	Johnston et al., 2020 [6]
	% testing positive for BRAF mutation	2.0%	Johnston et al., 2020 [6]
	% testing positive for V600E mutation among those with BRAF mutation ^a^	64.5%	Martos et al., 2021 [18]
	% testing positive for KRAS mutation	25.0%	Johnston et al., 2020 [6]
	% testing positive for G12C mutation among those with KRAS mutation ^a^	41.0%	Zer et al., 2015 [19]
	% testing positive for MET mutation ^a^	1.0%	Johnston et al., 2020 [6]
	% testing positive for HER2/ERBB2 mutation ^a^	3.0%	Johnston et al., 2020 [6]
	% testing positive for RET mutation ^a^	1.0%	Johnston et al., 2020 [6]
	% testing positive for NRG1 mutation ^a^	0.3%	Laskin et al., 2020 [20]
	% testing positive for NTRK1 mutation ^a^	0.1%	Ou et al., 2019 [21]
	% testing positive for NTRK2 mutation ^a^	0.01%	Ou et al., 2019 [21]
	% testing positive for NTRK3 mutation ^a^	0.01%	Ou et al., 2019 [21]
Rebiopsies	% needing rebiopsy after each test (NGS)	12.1%	Tomlins et al., 2021 [26]
	% needing rebiopsy after each test (single-gene testing strategies)	5.5%	VanderLaan et al., 2018 [27]
	% receiving rebiopsy after each test (NGS and single-gene testing strategies)	30.0%	Chu et al., 2020 [22]
	% failed rebiopsy (of each rebiopsy attempted)	12.0%	Lim et al., 2017 [28]
	% with rebiopsy complications	9.0%	Chu et al., 2020 [22]
Time-to-test and clinical turnaround time (weeks)	Time-to-test result, NGS	3.0	Makarem et al., 2021 [29]
	Time-to-test result, EGFR, ALK, ROS1 mutations, per mutation	1.0	Makarem et al., 2021 [29]
	Time-to-test result, KRAS	3.0	Makarem et al., 2021 [29]
	Time-to-test result, all other mutations, per mutation	1.5	Pennell et al., 2019 [12]
	Time-to-oncologist visit	2.0	Makarem et al., 2021 [29]
	Time-to-rebiopsy	2.0	Makarem et al., 2021 [29]
**Cost inputs**		**(2021 CAD) ^b^**	**Source**
Testing	NGS (tissue)	CAD 1000	Makarem et al., 2021 [29]
	EGFR ^c^	CAD 240	Makarem et al., 2021 [29]
	ALK, NRTK1/2/3, per mutation ^d^	CAD 100	Makarem et al., 2021 [29]
	ROS1, MET, RET, NRG1, per mutation ^e^	CAD 400	Makarem et al., 2021 [29]
	BRAF, KRAS, HER2/ERBB2, per mutation ^c^	CAD 200	Makarem et al., 2021 [29]
	PD-L1 ^d^	CAD 138	Johnston et al., 2020 [30]
Medical	Rebiopsy ^f^	CAD 368	Ontario Schedule of Benefits, 2021 [31]
	Rebiopsy complication	CAD 5411	Canadian Institute for Health Information, 2019 [32]
	Interventional radiology visit	CAD 73	Ontario Schedule of Benefits, 2021 [31]
	Oncologist visit	CAD 157	Ontario Schedule of Benefits, 2021 [31]
	Oncologist follow-up visit	CAD 105	Ontario Schedule of Benefits, 2021 [31]
	Estimated cost associated with delaying care (per week)	CAD 406	De Oliveira et al., 2017 [15]

**Abbreviations:** FISH = fluorescence in situ hybridization; LC = lung cancer; NGS = next-generation sequencing; NSCLC = non-small cell lung cancer; PCR = Polymerase Chain Reaction; UHN = University Health Network. **Notes**: a. Actionable mutation considered to have a Health Canada approved targeted therapy. b. Cost values were reported in 2021 CAD, inflated based on the healthcare services component of the Statistics Canada Consumer Price Index [33]. c. PCR single-gene test cost based on UHN Laboratory Medicine Program costs. d. Immunohistochemistry single-gene test cost based on UHN Laboratory Medicine Program costs. e. FISH single-gene test cost based on UHN Laboratory Medicine Program costs. f. Rebiopsy costs were based on core needle biopsies.

**Table 2:** Model output summary.

	NGS	Sequential	Exclusionary	Non-Comprehensive	Rapid Panel	All Four Single-Gene Strategies Combined ^a^
**Clinical outputs**						
Proportion of patients tested (%)	50.0	5.0	10.0	25.0	10.0	50.0
Total patients tested (n)	190.8	19.1	38.2	95.4	38.2	190.9
Proportion of patients tested positive for a mutation with an approved targeted therapy ^b^ (%)	38.0	31.7	29.3	20.3	34.4	26.1
Patients tested positive for a mutation with an approved targeted therapy ^b^ (n)	72.4	6.1	11.2	19.4	13.1	49.7
Number of visits ^c^ (n)	384.8	55.9	103.7	271.1	107.4	538.2
Number of visits per patient ^c^ (n)	2.0	2.9	2.7	2.8	2.8	2.8
Number of rebiopsies (n)	3.2	2.4	4.4	3.8	0.8	11.5
Number of rebiopsies per patient (n)	0.02	0.13	0.12	0.04	0.02	0.06
Time to initiation of appropriate targeted therapy, per patient ^d^ (weeks)	5.1	16.1	15.3	6.4	7.0	9.2
**Cost outputs**						
Total costs ^e^ (CAD)	270,117	48,088	88,680	111,135	110,869	358,773
Total costs per patient (CAD)	1416	2518	2321	1165	2902	1879
Total costs including estimated costs associated with delaying care (CAD)	663,998	172,740	325,963	357,245	219,275	1,075,222
Estimated costs per patient associated with delaying care ^f^ (CAD)	2064	6526	6212	2580	2838	3752
Total costs per patient including estimated costs associated with delaying care (CAD)	3480	9044	8533	3745	5740	5632

**Abbreviation:** NGS = next-generation sequencing. **Notes** a. The model outputs from each individual single-gene testing strategy were combined to present the sum of all single-gene testing strategies, which may be slightly different from the total of each component individually due to rounding. b. All patients testing positive for the following actionable mutations were considered to have a Health Canada approved targeted therapy: EGFR, ALK, ROS1, BRAF V600E subtype, KRAS G12C subtype, MET, HER2, RET, NRG1, NTRK1, NTRK2, and NTRK3. c. The number of oncologist and interventional radiologist visits were added to obtain the total number of visits per patient. It was assumed that patients who required rebiopsy received an interventional radiologist visit for the procedure. At the beginning and end of the testing sequence, patients were assumed to receive one oncologist visit to discuss the results of the biopsy test, and another oncologist visit to plan for appropriate targeted therapy initiation. All patients undergoing a single-gene testing strategy who tested negative for EGFR and ALK mutations received an additional oncologist visit before continuing the testing sequence. d. Time to appropriate targeted therapy was calculated as the sum of time to test results for genomic alterations, as well as clinical turnaround time. For patients undergoing a single-gene testing strategy who tested negative for EGFR and ALK mutations, a 2-week clinical turnaround time was incorporated to discuss next steps in the testing sequence. For patients requiring rebiopsy, a 2-week clinical turnaround time was incorporated to undergo the procedure. Across all testing strategies, a 2-week clinical turnaround time was incorporated at the end of the testing sequence, for patients to see an oncologist and discuss test results and the initiation of either targeted therapy or best supportive care. Among patients undergoing testing with rapid panel testing, the time to test results for each simultaneous panel were based on the alteration with the longest time to test results (i.e., EGFR and KRAS). e. Costs include gene testing costs, rebiopsy costs, costs for interventional radiologist and oncologist visits, and PD-L1 testing costs. f. Total costs include testing costs, testing-related medical and diagnostic costs, and estimated costs of delayed care. The estimated costs associated with delaying treatment were calculated as the time to initiation of appropriate targeted therapy times estimated weekly cost during the pre-diagnosis phase.

**Table 3:** Sensitivity analysis inputs.

	Input	Low Case	High Case	Sources
Clinical Event Rates	Proportion of patients needing a rebiopsy	9.7%	14.5%	Assumption ^b^
	Proportion of patients with rebiopsy complications	7.2%	10.8%	Assumption ^c^
	Rate of successful identification of EGFR mutation	7.0%	20.6%	Forsyth et al., 2020 [34]; Shiau et al., 2014 [35]
	Rate of successful identification of KRAS mutation	20.0%	36.1%	Assumption ^c^; Alwithenani et al., 2021 [36]
Testing Costs	NGS (tissue)	CAD 750	CAD1250	Assumption ^d^
	EGFR	CAD 200	CAD288	Makarem et al., 2021 [29]; Assumption ^e^
	KRAS	CAD 160	CAD 240	Assumption ^b^
Diagnostic Testing/Medical Costs	PD-L1	CAD 104	CAD 166	Makarem et al., 2021 [29]; Assumption ^f^
	Rebiopsy	CAD 294	CAD 442	Assumption ^b^
	Rebiopsy complication	CAD 4329	CAD 6493	Assumption ^b^
	Interventional radiology visit	CAD 58	CAD 88	Assumption ^b^
	Oncologist visit for consultation	CAD 126	CAD 188	Assumption ^b^
	Oncologist follow-up visit	CAD 84	CAD 126	Assumption ^b^
	Cost of delaying treatment (per week)	CAD 325	CAD 487	Assumption ^b^

**Abbreviations:** NGS = next-generation sequencing; SGT = single-gene testing; UHN = University Health Network. Notes: a. Assumed to be −5 percentage points of base case value (low case value) or +5 percentage points (high case value). b. Assumed to be −20% of base case value (low case value) or +20% (high case value). c. Assumed to be −20% of base case value (low case value) since Canadian estimates do not fall below 25%. d. Assumed to be −25% of base case value (low case value) or +25% (high case value). e. Price of single-gene test from UHN Laboratory Medicine Program costs [low case]; +20% to approximate ratio of low cost to base case [high case]. f. Price of immunohistochemistry test from UHN Laboratory Medicine Program costs [low case]; +20% to approximate ratio of low cost to base case [high case].

**Table 4:** Impact of increasing the proportion of patients tested with NGS.

	NGS	Sequential	Exclusionary	Non-Comprehensive Sequential	Rapid Panel	Total (All Strategies) ^a^
**Base case with 50% of patients tested by NGS**						
Proportion of patients tested (%)	50.0	5.0	10.0	25.0	10.0	100.0
Number of patients tested by strategy (*n*)	190.8	19.1	38.2	95.4	38.2	381.7
Proportion of patients tested positive for a mutation with an approved targeted therapy^2^ (%)	38.0	32.1	29.3	20.3	34.4	32.0
Patients tested positive for a mutation with an approved targeted therapy ^b^ (*n*)	72.4	6.1	11.2	19.4	13.1	122.3
Time to initiation of appropriate targeted therapy, per patient ^c^ (weeks)	5.1	16.1	15.3	6.4	7.0	7.2
Total annual cost at the plan level ^d^ (CAD)	270,117	48,088	88,680	111,135	110,869	628,890
Total annual cost at the plan level including estimated costs associated with delaying care ^e^ (CAD)	663,998	172,740	325,963	357,245	219,275	1,739,220
**Increased proportion with 70% of patients tested by NGS**						
Increased proportion of patients tested by NGS (%)	70.0	5.0	5.0	15.0	5.0	100.0
Number of patients tested by strategy (*n*)	267.1	19.1	19.1	57.2	19.1	381.7
Proportion of patients tested positive for a mutation with an approved targeted therapy ^b^ (%)	38.0	31.7	29.3	20.3	34.4	34.4
Patients tested positive for a mutation with an approved targeted therapy ^b^ (*n*)	101.4	6.1	5.6	11.6	6.6	131.3
Time to initiation of appropriate targeted therapy, per patient ^c^ (weeks)	5.1	16.1	15.3	6.4	7.0	6.5
Total annual cost at the plan level ^d^ (CAD)	378,135	48,240	44,498	66,641	55,435	592,796
Total annual cost at the plan level, including estimated costs associated with delaying care ^e^ (CAD)	929,528	172,740	162,981	214,197	109,637	1,589,084
**Incremental impact**						
Incremental patients tested positive for a mutation with an approved targeted therapy ^b^ (*n*)	29.0	0.0	−5.6	−7.8	−6.5	9.1
Incremental annual budget impact ^d^, total (CAD)	108,017	0	−44,183	−44,494	−55,435	−36,094
Incremental budget impact, PMPM (CAD)	0.009	0.000	−0.004	−0.004	−0.005	−0.003
Incremental annual budget impact, including estimated costs associated with delaying care ^e^, total (CAD)	265,530	0	−162,981	−143,048	−109,637	−150,137
Incremental budget impact, including estimated costs associated with delaying care, PMPM (CAD)	0.022	0.000	−0.014	−0.012	−0.009	−0.013

**Abbreviations:** NGS = next-generation sequencing; PMPM = per member per month. **Notes:** a. The combined sum of all the single-gene strategies may be slightly different from the total of each component individually due to rounding. b. All patients testing positive for the following actionable mutations were considered to have a Health Canada-approved targeted therapy: EGFR, ALK, ROS1, BRAF V600E subtype, KRAS G12C subtype, MET, HER2, RET, NRG1, NTRK1, NTRK2, and NTRK3. c. Time to appropriate targeted therapy was calculated as the sum of time to test results for genomic alterations, as well as clinical turnaround time. For patients undergoing a single-gene testing strategy who tested negative for EGFR and ALK mutations, a 2-week clinical turnaround time was incorporated to discuss next steps in the testing sequence. For patients requiring rebiopsy, a 2-week clinical turnaround time was incorporated to undergo the procedure. Across all testing strategies, a 2-week clinical turnaround time was incorporated at the end of the testing sequence, for patients to see an oncologist and discuss test results and the initiation of either targeted therapy or best supportive care. Among patients undergoing testing with rapid panel testing, the time to test results for each simultaneous panel were based on the alteration with the longest time to test results (i.e., EGFR and KRAS). d. Costs include gene testing costs, rebiopsy costs, costs for interventional radiologist and oncologist visits, and PD-L1 testing costs. e. Total costs include testing costs, testing-related medical and diagnostic costs, and estimated costs of delayed care. The estimated costs associated with delaying treatment were calculated as the time to initiation of appropriate targeted therapy times estimated weekly cost during the pre-diagnosis phase.

## Data Availability

The data underlying this article are available in the article. Some of the results were included in an oral presentation at the IASLC (International Association for the Study of Lung Cancer) 2022 WCLC (World Conference on Lung Cancer), held 6–9 August 2022, in Vienna, Austria.
